# Case series of topical 1.5% ruxolitinib cream for pediatric vitiligo

**DOI:** 10.1016/j.jdcr.2024.02.034

**Published:** 2024-04-18

**Authors:** Harry M. Meister, Mark Lebwohl, Nanette Silverberg

**Affiliations:** Department of Dermatology, Icahn School of Medicine at Mount Sinai, New York, New York

**Keywords:** Janus kinase inhibitors, mixed vitiligo, nonsegmental vitiligo, pediatrics, repigmentation, ruxolitinib, segmental vitiligo, treatment

## Introduction

Pediatric vitiligo affects 1.52% of 4 to 11-year-olds and 2.16% of 12 to 17-year-olds within the United States, with nonsegmental vitiligo (NSV) affecting 65% and 69%, respectively, and the residuum segmental vitiligo (SV).^1^ The Food and Drug Administration (FDA) has approved topical 1.5% ruxolitinib cream for NSV affecting 10% of body surface area or less for 12 years of age or older. Although the TRuEV-1 and TRuEV-2 trials included children as young as 12 years, the data for the subset of children was not separately analyzed in publication.^2^ Furthermore, there is no FDA-approved topical agent for SV in any age group.^3^ We sought to characterize the efficacy of 1.5% ruxolitinib cream for pediatric vitiligo.

## Methodology

An IRB-exempted chart review was conducted addressing the usage of 1.5% ruxolitinib cream in children with vitiligo of all types (0-17 years of age) who used topical 1.5% ruxolitinib cream for the treatment of vitiligo. The patients were treated according to standard protocol for NSV of twice a day, however, 2 patients reduced to daily application, and 308-nm laser was recommended concurrently for all segmental cases.

## Case series

Twelve children were identified, ages 3 to 16 years (average age: 10.75 years).

Demographics included males (*n* = 9), females (*n* = 3). Race/ethnicity data included children who were Black (*n* = 1), Hispanic/Latin X (*n* = 4), Indian (*n* = 1), and White (*n* = 6).

The vitiligo subtypes included NSV (*n* = 7), mixed-type vitiligo which is a combination of SV and NSV (*n* = 1), and isolated SV (*n* = 4). The summary of these cases is shown in Table I.

**Table I:** Summary of 12 children treated for vitiligo with topical ruxolitinib 1.5% cream

Case	Type of vitiligo	Age (y)	Sex Race Ethnicity	Location lesions	Time with vitiligo (mo)/frequency therapy	Time until first response (mo)	Add-on therapies	Laboratory tests	Repigmentation, %	Time to final response (mo)
1	NSV	3	Male Indian	Forehead, perioral	5/every day	3	None	CBC TFT WNL	100%	5
2	NSV	9	Female Black	Perioral, upper portion of the right arm, right calf	4/twice a day	1	None	CBC CMP TFT WNL	100%	9
3	NSV	9	Male Hispanic	Bilateral thighs, knees, calves	4/twice a day	2	None	CBC CMP WNL	100%	6
4	NSV	10	Male Hispanic	Bilateral hands, thighs, knees, calves, ankles	14/twice a day	1	None	None	100%	5
5	NSV	12	Male Hispanic	Periocular, perioral, bilateral hands, thighs, knees, calves	1/twice a day	Partial repigmentation at 3 mo/premature discontinuation	None	CBC CMP WNL	70%	3
6	NSV	15	Female White	Bilateral upper portion of the arms, forearms, hands, thighs, knees, calves	4/twice a day	2	None	None	100%	5
7	NSV	15	Male White	Face, hands	5/every day	3	None	CBC CMP WNL	100%	6
8	Mixed	10	Female Hispanic	Knees (NSV)	12/twice a day	3	NB UVB	None	100%	12
				Lower portion of the abdomen/left leg (SV)	72/twice a day	No repigmentation				
9	SV	5	Male White	Neck	3/twice a day	No repigmentation/hair color preserved	None	None	0%	6 mo trial
10	SV	11	Male White	Chest, back	3/twice a day	6	NB UVB	None	70%	6 mo trial
11	SV	14	Male Male	Right perioral,right chin	1/twice a day	1	NB UVB Acne meds	None	95% (persistent poliosis)	9
12	SV	16	Male White	Scalp, left side of the forehead/left periocular/left cheek	1/twice a day	1	Dexamethasone pulsed therapy and NB UVB	CBC CMP TSH WNL	99% (persistent poliosis)	5

*CBC*, Complete blood count; *CMP*, complete metabolic profile; *NB UVB*= narrowband ultraviolet B phototherapy; *NSV*, nonsegmental vitiligo; *SV*, segmental vitiligo; *TFT*, thyroid function tests; *TSH*, thyroid stimulating hormone; *UVB*, ultraviolet B phototherapy; *WNL*, within normal limits.

**Fig 1:**
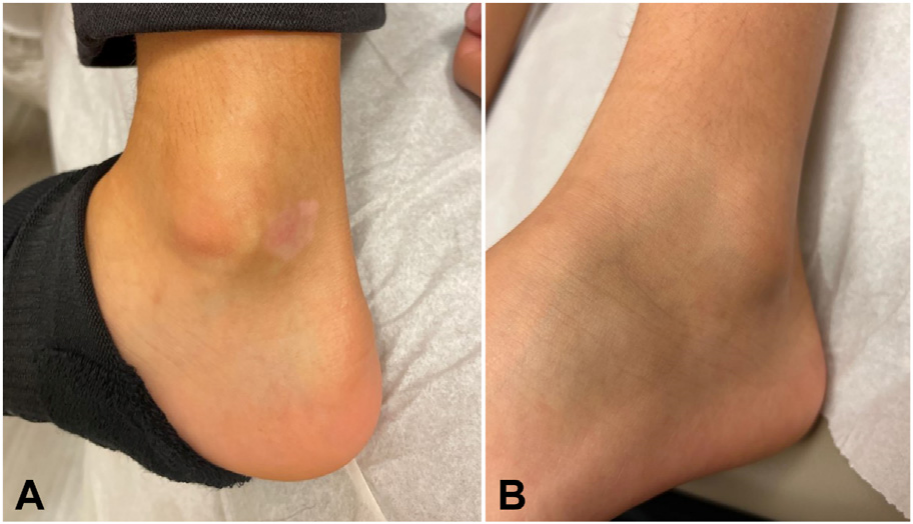
**A, B,** Patient 4 before and after therapy.

The NSV group and the nonsegmental component of the mixed type included 8 children, average age of 10.4 years (range 3-15 years). Treatment was once daily (*n* = 2) or twice-daily (*n* = 6). Average maximum repigmentation occurred at 6.4 months (range 3-12 months), with 6 achieving complete repigmentation and 1 with 70% repigmentation after 3 month trial. NSV was present on average for 6.1 months (1-12 months). Patient 4 is demonstrated in Fig 1 before and after treatment of the ankles. Onset of response was 2.25 months on average (range 1-3 months), starting on the face (*n* = 4), arms (*n* = 2), and abdomen (*n* = 1). No adverse events were reported. Five children had normal complete blood counts while on medication. Four patients previously failed tacrolimus topically, 2 patients failed topical class 2 corticosteroids, and 1 previously failed narrowband ultraviolet B light.

**Fig 2:**
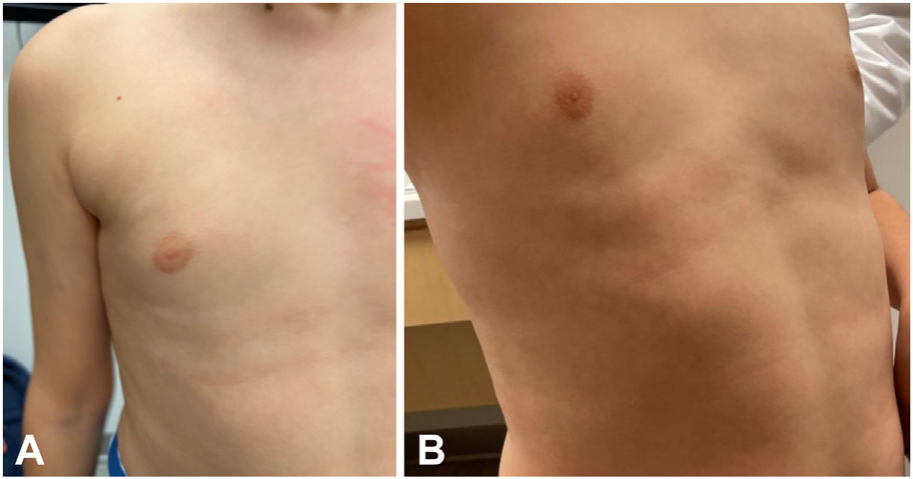
**A, B,** Patient 10 before and after therapy.

There were 5 patients with SV average age 11.2 years (range 5-16 years) including the segmental component of the mixed disease patient with facial (*n* = 1), face/neck (*n* = 1), neck (*n* = 1), chest/back (*n* = 1), and lower portion of the abdomen (*n* = 1). One 5-year-old neck SV failed to repigment after 6 months, but did not develop poliosis. A 10-year-old with segmental disease for 6 years had no response. One patient responded who had failed topical tacrolimus and class 2 topical corticosteroids (4 month trial). Maximal repigmentation required concurrent 308-nm laser for the facial SV (12 treatments), chest/back SV (12 treatments), and 1 NSV had concurrent 308-nm laser (24 treatments) with pulsed dexamethasone, without any notable adverse events. Two teenage males developed acne on sites of application on the face. Patient 10 is demonstrated in Fig 2 before and after treatment of the ankles laboratory screening including complete blood count was normal during treatment for 2 patients evaluated.

## Discussion

There is a growing body of evidence that the topical JAK inhibitor ruxolitinib 1.5% cream may be beneficial in selected cases of pediatric inflammatory skin disease. Data has been published from a recent placebo-controlled clinical trial of topical ruxolitinib 1.5% cream for pediatric atopic dermatitis in children ages 2 to 17 years. The patients had very few, mild adverse events, bone markers were not altered, and blood levels were very low, however, long-term data is lacking for both pediatric atopic dermatitis and vitiligo.^3^

In pediatric vitiligo, the FDA has approved ruxolitinib 1.5% cream twice a day for vitiligo in 12 years of age and older, for 10% or less body surface area, based on 2 phase 3 clinical trials, the TRuEV-1 and TRuEV-2 trials.^2^ Notably the trajectory of repigmentation is faster onset and rapid completion in children with NSV in our case series. We believe that part of the reason for the faster and more complete response is that therapy was instituted rapidly in the patients with NSV. Additionally, although only NSV has FDA approval, we note partial repigmentation with rapid institution of topical ruxolitinib 1.5% cream twice-daily and the addition of 308-nm laser treatments. Finally, we noted 2 cases of acne in teenagers, but otherwise no local adverse events and no laboratory abnormalities where applicable. Two patients had excellent response and we believe daily dosing can be considered in children to reduce drug exposure.

Limitations of this study are that the patients were neither randomized nor placebo-controlled, and we did not have blood levels of medication.

Although more systematic, randomized data is needed in children ages 2 to 11 years with vitiligo, it appears that the early institution of topical 1.5% ruxolitinib cream promotes rapid onset of repigmentation and rapid complete response in both NSV, and in the first 6 months of SV. This safety profile appears to be similar to that of recently presented data in children 2 to 11 years with atopic dermatitis, but the sample size is too small to make strong conclusions.^4^ Vehicle-controlled clinical trials of 1.5% topical ruxolitinib with and without narrowband ultraviolet B light sources for NSV and SV are needed to identify outliers and long-term outcomes.

## Conflicts of interest

Dr Silverberg is a consultant, adviser, or speaker for Amryt, Incyte, Sanofi-Regeneron, Novan, and Verrica. Dr Lebwohl is an employee of Mount Sinai and receives research funds from AbbVie, Amgen, Arcutis, Avotres, Boehringer Ingelheim, Cara therapeutics, Dermavant Sciences, Eli Lilly, Incyte, Inozyme, Janssen Research & Development, LLC, Ortho Dermatologics, Pfizer, Sanofi-Regeneron, and UCB, Inc, and is a consultant for Almirall, AltruBio Inc, AnaptysBio, Apogee, Arcutis, Inc, AstraZeneca, Atomwise, Avotres Therapeutics, Brickell Biotech, Boehringer Ingelheim, Bristol-Myers Squibb, Castle Biosciences, Celltrion, CorEvitas, Dermavant Sciences, EPI, Evommune, Inc, Facilitation of International Dermatology Education, Forte biosciences, Foundation for Research and Education in Dermatology, Galderma, Genentech, Incyte, LEO Pharma, Meiji Seika Pharma, Mindera, Pfizer, Sanofi-Regeneron, Seanergy, Strata, Takeda, Trevi, and Verrica. Author Meister has no conflicts of interest to declare.
